# A Novel Liquid Biopsy Method Based on Specific Combinations of Vesicular Markers Allows Us to Discriminate Prostate Cancer from Hyperplasia

**DOI:** 10.3390/cells13151286

**Published:** 2024-07-31

**Authors:** Emanuele Martorana, Gabriele Raciti, Raffaella Giuffrida, Elena Bruno, Vincenzo Ficarra, Giuseppe Mario Ludovico, Nazareno Roberto Suardi, Nunzio Iraci, Loredana Leggio, Benedetta Bussolati, Cristina Grange, Aurelio Lorico, Rosario Leonardi, Stefano Forte

**Affiliations:** 1 IOM Ricerca Srl, Viagrande, 95029 Catania, Italy; emanuele.martorana@grupposamed.com (E.M.); gabriele.raciti@grupposamed.com (G.R.); raffaella.giuffrida@grupposamed.com (R.G.); alorico@touro.edu (A.L.); 2 Department of Biomedical, Dental and Morphological and Functional Imaging Sciences, University of Messina, 98122 Messina, Italy; 3 Department of Physic and Astronomy “Ettore Majorana”, University of Catania, 95123 Catania, Italy; elena.bruno@dfa.unict.it; 4 Azienda Ospedaliera Policlinico Universitario “G. Martino”, Dipartimento di Patologia Umana dell’Adulto e dell’Età Evolutiva, 98124 Messina, Italy; vficarra@unime.it; 5 Ospedale Generale Regionale “F. Miulli”, Divisione di Urologia, Acquaviva Delle Fonti, 70021 Bari, Italy; giuseppeludovico@hotmail.com; 6 Azienda Ospedaliera Policlinico Universitario Di Genova, Divisione di Urologia, 16132 Genova, Italy; suardi.nazareno@gmail.com; 7 Department of Biomedical and Biotechnological Sciences, University of Catania, 95123 Catania, Italy; nunzio.iraci@unict.it (N.I.); loredana.leggio@unict.it (L.L.); 8 Department of Molecular Biotechnology and Health Sciences, University of Turin, 10124 Turin, Italy; benedetta.bussolati@unito.it; 9 Department of Medical Sciences, University of Turin, 10124 Turin, Italy; cristina.grange@unito.it; 10 College of Osteopathic Medicine, Touro University Nevada, Henderson, NV 89014, USA; 11 Casa di Cura Musumeci-Gecas, 95030 Gravina di Catania, Italy; leonardi.r@tiscali.it

**Keywords:** prostate cancer, extracellular vesicles, biomarkers, liquid biopsy

## Abstract

Background: Prostate cancer is the second most common cancer in males worldwide, and its incidence is rising. Early detection is crucial for improving the outcomes, but the current screening methods have limitations. While prostate-specific antigen (PSA) testing is the most widely used screening tool, it has poor specificity, leading to a high rate of false positives and unnecessary biopsies. The existing biopsy techniques are invasive and are associated with complications. The liquid biopsy methods that analyze the biomarkers in blood or other bodily fluids offer a non-invasive and more accurate alternative for detecting and characterizing prostate tumors. Methods: Here, we present a novel liquid biopsy method for prostate cancer based on the identification of specific proteins in the extracellular vesicles isolated from the blood of patients with prostate cancer. Results: We observed that a specific combination of sEV proteins is a sensitive indicator of prostate cancer. Indeed, we found that the number of clusters expressed by specific combinations of either intra-vesicular (STAT3 and CyclinD1) or surface proteins (ERBB3, ALK, and CD81) allowed us to significantly discriminate the patients with prostate cancer from the individuals with hyperplasia. Conclusion: This new liquid biopsy method has the potential to improve prostate cancer screening by providing a non-invasive and more accurate diagnostic tool.

## 1. Introduction

According to the Global Cancer Observatory (GCO) of the International Agency for Research on Cancer (IARC), prostate cancer (PCa) is the second most commonly diagnosed cancer in men worldwide, after lung cancer. In 2020, there were an estimated 1.4 million new cases of PCa, accounting for 7.3% of all the new cancer cases in men. The incidence of PCa varies widely by region, with the highest rates observed in developed countries, such as in North America, Europe, and Australia, and lower rates observed in developing countries in Asia and Africa [1,2].

The early and accurate diagnosis of PCa is crucial for effective treatment and improved patient outcomes [3,4]. Over the years, various diagnostic approaches have been employed to detect and characterize PCa, aiming to strike a balance between the identification of aggressive tumors requiring intervention and the avoidance of unnecessary treatments for indolent tumors.

The diagnosis of PCa traditionally involves a combination of screening, clinical evaluation, and confirmatory tests. Prostate-specific antigen (PSA) testing has been widely utilized as a screening tool due to its convenience and availability [5,6]. However, PSA testing has limitations in terms of specificity, leading to a considerable number of false positive results and subsequent invasive procedures such as biopsies [7,8,9]. To address this, additional clinical parameters, such as digital rectal examination (DRE) and patient-specific factors (e.g., age and family history), are considered in the decision-making process.

Confirmatory tests, such as transrectal ultrasound (TRUS)-guided biopsy, have long been the gold standard for diagnosing PCa. This invasive procedure involves obtaining small tissue samples from the prostate gland for pathological examination. However, TRUS-guided biopsy is not without limitations. It may miss small or hard-to-reach tumors, resulting in false negative results and delayed diagnosis. Moreover, the procedure carries potential risks, including infection and bleeding, emphasizing the need for alternative diagnostic approaches [10].

Advances in medical technology and the understanding of PCa biology have led to the development of novel diagnostic methods. Magnetic resonance imaging (MRI) has emerged as a valuable tool in PCa diagnosis, providing detailed images of the prostate gland and aiding in the detection and characterization of suspicious lesions. Furthermore, MRI fusion-guided biopsies have shown promise in improving the accuracy of targeted biopsies, minimizing unnecessary procedures and better identifying clinically significant tumors. While magnetic resonance imaging (MRI) is a valuable tool in the diagnosis and evaluation of PCa, it also has certain limitations, such as dependency on an operator to obtain results [11,12], costs [12], and availability [13].

Liquid biopsy, a non-invasive diagnostic approach, has gained significant attention in recent years. By analyzing various biomarkers, such as circulating tumor cells (CTCs), circulating cell-free DNA (cfDNA), and extracellular vesicles (EVs), liquid biopsies offer the potential for early cancer detection, monitoring treatment responses, and assessing tumor heterogeneity. This approach may revolutionize PCa diagnosis by providing a more precise, timely, and affordable assessment of disease in the initial phase. In particular, EVs may be considered a consistent source of tumor-derived biomarkers due to their prevalence in body fluids and to the stability offered by the phospholipid bilayer that protects the tumor-originated molecules from degradation [14]. Some different examples of EV- based analysis have been proposed for PCa [15,16,17,18,19]. Both blood [17,18,19] and urine [15,16] EVs have been investigated for their potential role in PCa diagnosis. While both the EV sources can be potentially used in the clinics, blood and urine EVs have different features that may impact their actual usage in different clinical setting for PCa diagnosis and management. Blood-based liquid biopsy methods offer significant advantages over urine-based approaches, particularly in the realm of early cancer diagnosis protocols [20]. One of the key advantages lies in the stability of tumor markers in blood samples, a characteristic that remains consistent regardless of the time of day the sample is collected. The reliability of blood-based methods, in fact, is underscored by the absence of fluctuations caused by external factors, such as hydration, diet, or medication, which can significantly impact the composition of urine samples and lead to variability in tumor marker concentrations. The EV cargo comprises lipids, proteins, and nucleic acids (both DNA and several types of RNA) [21,22,23]. These molecules are specifically associated with the EVs produced by cells. Vesicles are thus diffusible elements representing, at least in part, the molecular landscape of the cells that release them. There are many molecular pathways that have been specifically associated with prostate cancer. While many of the proteins related to these pathways failed to be used as target for therapeutic intervention, their significant modulation in PCa suggests a possible use as biomarkers for early diagnosis. HERBB3, ALK, STAT3, and CyclinD1 are among the most commonly investigated proteins involved in PCa dynamics. HERBB3 is one of the principal activators of HER2 according to its ability to heterodimerize upon the binding of a large number of ligands. It has been demonstrated that ERBB signaling is implicated in CRPC [24]; nevertheless, clinical trials of ERBB-targeting therapies failed to demonstrate antitumor activity [25]. It is very important to note that unlike other tumors, genomic aberrations in the ERBB/HER genes are uncommon in prostate cancer [26]. This indicates that augmented expression, rather that mutagenic activation, is a specific feature that must be considered when using ERBB3 as a potential biomarker for PCa. In a similar way, it has been suggested that an increased level of ALK activity, which also involves CyclinD1, is strongly associated to the acquisition of an aggressive phenotype in the prostate cells [27,28]. Another common hallmark of prostate carcinogenesis is also represented by STAT3 signaling activation [29].

Differently from other members of the tetraspanins family, the role of CD81 in cancer development has been only recently explored more in detail. It is involved in solid tumors, including in hepatocellular carcinoma [30], gastric cancer [31], breast cancer [32,33,34], osteosarcoma [35], and melanoma [36], even though it acts in an apparently contradictory fashion. However, most researchers agree in depicting its deregulation that is prone to triggering tumor progression both in vitro and in vivo, so it has been successfully tested as a therapeutic target in breast cancer [33]. Moreover, human tissue analysis itself has displayed a closer correlation between CD81 overexpression and tumor specimens compared to that of the non-malignant ones [36,37]. Altogether, such evidences make CD81 deserving of further investigations, particularly in PCa, where has been just partially explored [37].

In this work, we investigated the feasibility of the blood-based liquid biopsy approach for diagnosis support in suspected early-onset PCa. The method is based on the evaluation of specific blood sEV protein markers assessed by super-resolution microscopy. This method is reproducible and sensitive enough to precisely estimate the number of single-, double- and triple-positive EVs, and a specific algorithm has been developed to discriminate the tumor samples from the hyperplasia ones.

## 2. Materials and Methods

### 2.1. Patient Enrollment, Blood Samples, and Plasma Isolation

This study received ethical approval from the “Catania 2” ethical board (protocol number 699/C.E. of 18/11/2020) and from all the ethical review boards of each participating center. The inclusion criteria were being aged ≥ 18 and having a PSA between 2.5 and 10 ng/mL, a definitive diagnosis of prostate adenocarcinoma (PCa) (for the prostate cancer group) or benign prostatic hyperplasia (BPH) (for the BPH group), no evidence of metastasis, and a SARS-CoV-2 negative status. Written informed consent was obtained from each patient before any study-related assessments. The exclusion criteria included patients with tumors other than prostate cancer and patients with viral pathologies, such as hepatitis, HIV infections, or other conditions, that could affect the study results or compromise the safety of the procedures. A total of 54 individuals (42 patients with PCa and 12 individuals with BPH) were enrolled in this multicenter study involving five participating prostate cancer centers.

Blood samples, each containing 10 mL, were collected from all the participating individuals using RNA Complete BCT tubes (Streck, La Vista, NE, USA) to prevent the degradation of extracellular vesicles (EVs). These samples were then sent from each participating recruitment center to the central laboratory for further processing.

Upon arrival at the central laboratory, the samples were centrifuged at room temperature (RT) for 10 min at 1.600× *g* to remove the cellular components. The resulting supernatant, referred to as plasma, was carefully separated from the cell pellet using a Pasteur pipette. Subsequently, the plasma was subjected to a second centrifugation step for 10 min at 3000× *g* to eliminate any remaining cell debris.

The processed plasma samples were divided into 1-milliliter aliquots and stored at a freezing temperature of −80 °C for subsequent analyses.

### 2.2. Extracellular Vesicle Isolation

For the isolation of extracellular vesicles (EVs), 1 mL single aliquots of frozen plasma samples were rapidly thawed at 37 °C, and then immediately placed on ice for the subsequent procedures. The samples were diluted in a 1:1 ratio with cold PBS, and then underwent sequential centrifugation conducted at 4 °C.

First, the diluted plasma was centrifuged for 10 min at 4000 rpm to remove any remaining cells. The resulting supernatant was subjected to further centrifugation for 30 min at 10.500 rpm to eliminate cellular debris and large EVs. Before proceeding with ultracentrifugation (UC), the plasma was filtered through a 0.22 μm filter to eliminate any impurities.

Small EVs were isolated through ultracentrifugation (60 min, 200,000× *g*) using a Sorvall Discovery 90SE ultracentrifuge equipped with a Thermo Scientific (Waltham, MA, USA) TH-660 Titanium swinging bucket rotor. Subsequently, the sEVs were washed with PBS and underwent another ultracentrifugation step lasting 60 min at 200,000× *g*.

The resulting pellet was resuspended in 100 μL of cold PBS and stored at −80 °C. For the quantification of sEV-associated proteins, 10 μL of the EV suspension was used in accordance with the Pierce BCA protein assay kit (Thermo Scientific) protocol. Absorbance was measured using a spectrophotometer at 562 nm, and the protein concentration was determined using a BSA standard curve.

### 2.3. Extracellular Vesicle Characterization

#### 2.3.1. Nanoparticle Tracking Analysis

The size and concentrations of EVs derived from both the PCa and BPH plasmas were determined by Nanoparticle Tracking Analysis (NTA) using ZetaView (software version: 8.05.10; Particle Metrix GmbH, Meerbusch, Germany) according to the manufacturer’s protocol.

#### 2.3.2. Scanning Electron Microscopy (SEM)

EV morphology was studied using a scanning electron microscope Gemini Field Emission SEM Carl Zeiss SUPRATM 25 (FEG-SEM, Carl Zeiss Microscopy GmbH, Jena, Germany) in Inlens mode using a 3 kV electron beam. SEM samples were prepared as follows: the EVs recovered from 200,000 g pellet were resuspended in 500 μL di H_2_O, and then a 50 μL aliquot of EV suspension was dropped onto pin stubs coated with PELCO carbon conductive tabs and let dry at room temperature.

#### 2.3.3. Western Blot (WB)

Jurkat cells and EV extracts were processed as reported by Leggio et al. [38]. Briefly, the cells and EVs were lysed in RIPA buffer (10 mM Tris HCl pH 7.2 (Fisher Scientific, Hampton, NH, USA, BP152); 150 mM NaCl (Sigma Aldrich, St. Louis, MO, USA, S7653); 1% Sodium deoxycholate (Sigma Aldrich, 30970); 0,1% (for cells) or 3% (for EVs) SDS (Sigma Aldrich, 71736); 1% Triton X-100 (Sigma Aldrich, T8787); 1 mM EDTA pH 8 (VWR chemicals, Avantor, Radnor, PA, USA, E177-100ML); 1X Complete Protease inhibitor cocktail (Roche, Basel, Switzerland, 04693116001); and 1 mM Phenylmethanesulfonyl fluoride solution (PMSF, Sigma Aldrich, 93482). Totals of 7 µg of Jurkat cell lysate and 30 µg of EV lysates were loaded into 4–12% Bis-Tris plus gels (Invitrogen, Waltham, MA, USA, NW04125BOX) in reducing conditions. The proteins were transferred onto a PVDF membrane. All the primary and secondary antibodies are listed in Table 1. Jurkat cells were used as the positive control for CD45.

#### 2.3.4. Super-Resolution Microscopy

Three-dimensional (3D) direct stochastic optical reconstruction microscopy (dSTORM) was employed for the analysis of extracellular vesicles (EVs) using the Nanoimager S Mark II microscope from ONI (Oxford Nanoimaging, Oxford, UK). This microscope was equipped with a 100×, 1.4 NA oil immersion objective, an XYZ closed-loop piezo 736 stage, and three emission channels split at 473/488 nm, 561 nm, and 640 nm.

The experiments were conducted using the EV Profiler Kit from ONI (product code: EV-MAN-1.0, Oxford Nanoimaging, Oxford, UK) following the manufacturer’s protocol. In brief, the assay chip’s surface was initially activated by applying 5 µL of Surface Solution S3 to each lane and incubating for 10 min at room temperature (RT). Afterward, excess S3 was removed by washing with W1 Wash Solution, and then 10 µL of S4 Surface Solution was pipetted into each lane and incubated for an additional 10 min at RT.

The EV capture process was carried out as follows: 6 µL of sEV suspension was added to a mixture consisting of 3 µL of W1 and 1 µL of C1 Capture Supplement, resulting in a final reaction volume of 10 µL. This mixture was then distributed among the lanes and incubated for 30 min at RT to capture the EVs. The unbound sEVs were subsequently removed by washing with W1, followed by 10 min incubation with 20 µL of F1 Fixation Solution at RT.

After washing, each lane underwent 10 min incubation with 10 µL of either P1 solution (for permeabilization and blocking) or N1 solution (for blocking) to facilitate the staining of intra-vesicular or surface targets, respectively. As per the recommendations, an initial working solution with a 1:20 dilution of each antibody in W1 was prepared. A total of 1 µL of this solution was added to 9 µL of either P1 or N1 solution to achieve a final dilution of 1:200. Subsequently, 10 µL of the antibody solution was added to each lane and incubated for 50 min at RT, with the chip protected from light.

Following another washing step, 20 µL of F1 was applied and incubated for 10 min. Finally, 50 µL of BCubed imaging buffer was added to each lane, and image acquisition was immediately initiated.

Three channels (640 nm, 561 nm, and 473/488 nm) of dSTORM data were sequentially acquired at 30 Hz in total reflection fluorescence (TIRF) mode, with four acquisitions for each sample. Bead slide calibration was performed before each imaging session to align the fluorescent channels, ensuring a channel mapping precision smaller than 12 nm.

All the images were analyzed using algorithms developed by ONI via their CODI website platform https://alto.codi.bio/ (accessed on 14 February 2023) to minimize background noise and remove low-precision and non-specific co-localization. sEV population phenotypes (triple-, double-, or single-positive for each channel) and the number of clusters for each phenotype were determined and analyzed separately.

All the described reagents were supplied in the kit, including the fluorescent antibodies anti-CD9-CF^®^488A, anti-CD63-CF^®^568, and anti-CD81-CF^®^647. In addition, P1/N1 buffers from EV Profiler 2 Kit (product code: EV-PROFILER-2.0, Oxford Nanoimaging, Oxford, UK) were kindly supplied separately by the ONI company. Additionally, custom antibodies, such as anti-ErbB-3/HER3-AF^®^555 (Bioss Antibodies, bs-1454R-A555), anti-ALK-AF^®^488 (Bioss Antibodies, bs-0097R-A488), anti-STAT3-AF^®^555 (Bioss Antibodies, bs-3429R-A555), and anti-Cyclin D1-AF^®^488 (Bioss Antibodies, bs-0623R-A488), were used.

### 2.4. Statistical Analysis

The intra-vesicular and surface targets raw counts were first normalized through log2 scaling in order to reduce the range of data and make their distribution more symmetric. Welch’s *t*-test was used for the quantitative comparison of inter-sample means. Contingency tables and receiver operating characteristic (ROC) curves were used to evaluate the biomarkers classification performances. K-means was used as unsupervised learning technique to identify clusters in our dataset. The number of clusters for K-means was chosen using the Elbow method by selecting the best value that minimizes the within-cluster sum of squares. Benjamini–Hochberg correction was used for post-hoc analysis. The results were considered statistically significant when the *p*-value was less than 0.05. The data were analyzed using R (v4.2.2) [39] and R studio (v2022.12.0 Build 353) [40] to compare both the single and combined intra-vesicular or surface targets. The employed packages were ROCR [41] for the analysis of ROC curves, ggpubr [42], and ggplot2 [43] for data visualization.

## 3. Results

### 3.1. EV Characterization

**Figure 1:**
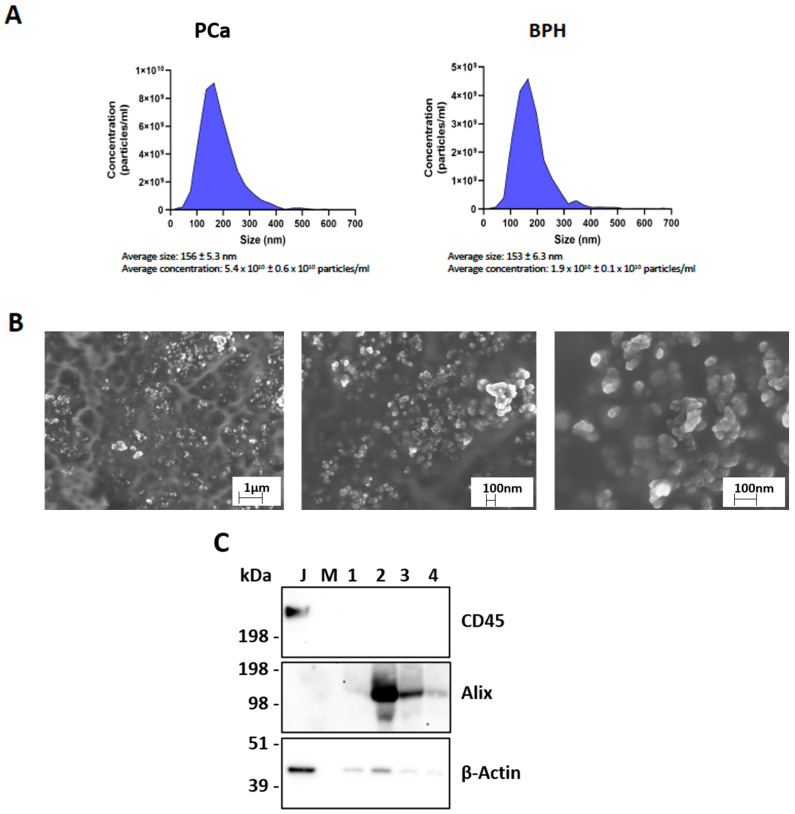
EV characterization. (**A**) Nanotracking analysis of PCa and BPH samples showing EV size and concentration. (**B**) Representative images of sEV acquisitions by SEM at different magnifications. (**C**) Western Blot analysis on Jurkat (J) and vesicular lysates (1, 2, 3, and 4) to detect CD45, Alix, and β-actin. Abbreviations: PCa: prostate cancer; BPH: benign prostate hyperplasia; sEVs: small extracellular vesicles; SEM: scanning electron microscopy; J: Jurkat cell lysate; M: marker.

The sEV pellets isolated from all the patients with BPH and PCa, whose clinical characteristics are reported in Table 2, underwent characterization for size and concentration using Nanoparticle Tracking Analysis (NTA) to ensure their suitability for dSTORM analysis. No significant differences in vesicle size and concentration were observed between the two sample types. In fact, as reported in Figure 1A, the size and concentration of EVs derived from both the BPH and PCa plasma were around 150 nm and 1.9 × 10^10^ and 5.4 × 10^10^ particles/mL, respectively. Moreover, further characterization using scanning electron microscopy revealed a moderate level of homogeneity in terms of morphology and size. All the samples exhibited the enrichment of vesicle populations, with an average size of approximately 100 nm (Figure 1B).

Additionally, as highlighted by Western Blot analysis, the sEV pellets from both the representative PCa (1 and 2 in Figure 1C) and BPH (3 and 4) samples expressed different concentration of the EV lumen protein Alix, sustaining its vesicular nature and intracellular origin. Moreover, the absence of CD45 in the EV lysates confirmed the absence of hematopoietic contamination that may arise from blood EV isolation. On the contrary, as expected, CD45 is significantly expressed in the Jurkat cells, an immortalized line of human T lymphocyte cells, lysate.

### 3.2. Selected Vesicular Marker Expression Allows Us to Discriminate PCa from BPH Samples

**Figure 2:**
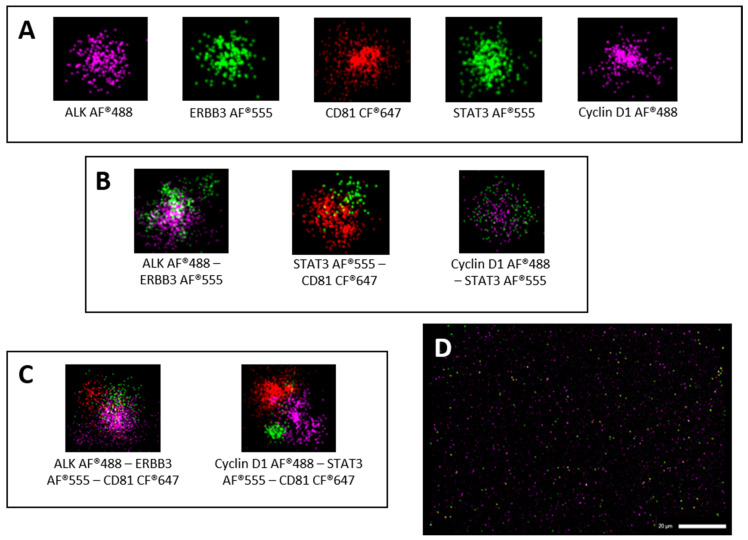

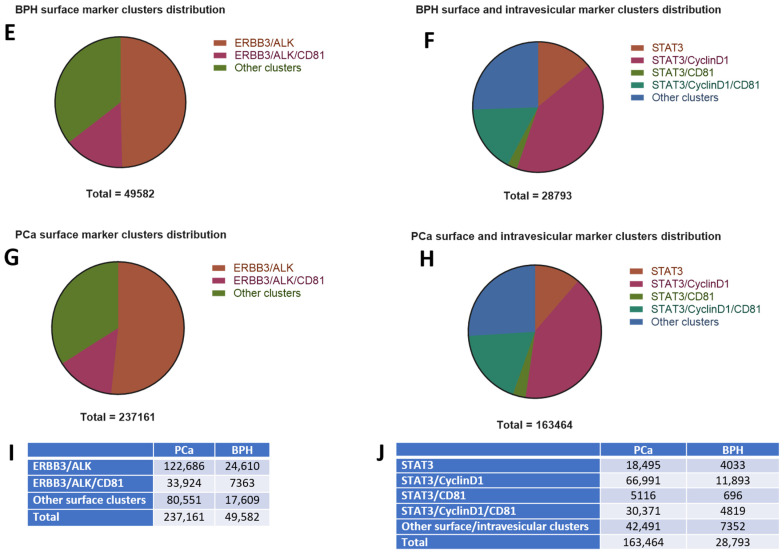
Super-resolution microscopy-based EV characterization. Representative images of single sEVs expressing only one (single labelling) of the analyzed markers (**A**) and co-expressing two (**B**) or three (**C**) markers contemporarily (double and triple labelling, respectively). Super-resolution microscopy field of view of sEVs isolated from PCa sample with scale bar of 20 µm (**D**). Pie charts of BPH surface-only (**E**) and surface/intra-vesicular (**F**) markers distribution and of PCa surface-only (**G**) and surface/intra-vesicular (**H**) ones. Surface-only (**I**) and surface/intra-vesicular (**J**) cluster counts for both types of all analyzed samples. Abbreviations: sEVs: small extracellular vesicles; PCa: prostate cancer; BPH: benign prostate hyperplasia; AF: alexa fluor dye; CF: cyanine-based far red fluorescent dye.

Super-resolution microscopy was also used to study the expression of selected surface (CD81, ERBB3, and ALK) and intra-vesicular proteins (STAT3 and CyclinD1) in the sEVs isolated from the plasma of all the patients with PCa and BPH (Figure 2). Figure S1 in the Supplementary Materials shows representative super-resolution acquisitions, pie charts of distribution, and cluster counts for those markers, which were not significant in the following experiments.

**Figure 3:**
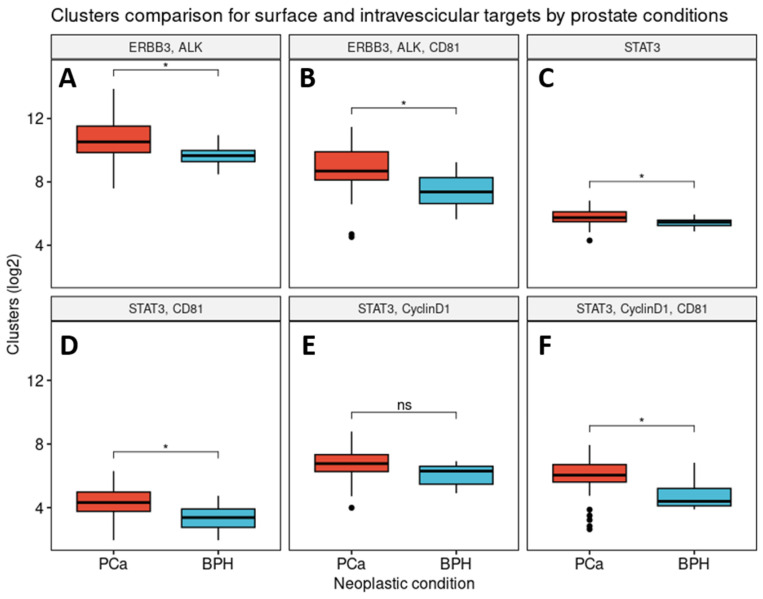
The cluster counts in the tumor and hyperplasia samples for the surface and intra-vesicular targets. The subfigures represent clusters expressing ERBB3 and ALK (**A**); ERBB3, ALK, and CD81 (**B**); STAT3 (**C**); STAT3 and CD81 (**D**); STAT3 and CyclinD1 (**E**); and STAT3, CyclinD1, and CD81 (**F**). Abbreviations: PCa: prostate cancer; BPH: benign prostate hyperplasia. Symbols represent: “*” *p* < 0.05; “ns” *p* ≥ 0.05; “•”refer to values out of ±1.5 * IQR.

The quantitative comparison of the number of clusters expressed by intra-vesicular and surface proteins was conducted individually or in coexistence. The cluster counts for intra-vesicular proteins show the statistically significant overexpression (*p* < 0.05) of STAT3 in the tumor group compared to that in the hyperplasia one (Figure 3C). Moreover, the other markers show significant differences when associated with STAT3 (Figure 3D–F), except for STAT3 and CyclinD1 (Figure 3E), where the difference is not significant. Surface proteins such as ERBB3 and ALK, both alone and combined with CD81 (Figure 3A,B) show significant differences in their expression within the two different sample types. In contrast, the other sEV proteins showed no significant different expression between the PCa and BPH samples (Figure S2). The comparison of PSA values between the subjects with PCa and BPH is not statistically significant *p* = 0.22 (Figure S6A), confirming that PSA does not reflect the group conditions.

**Figure 4:**
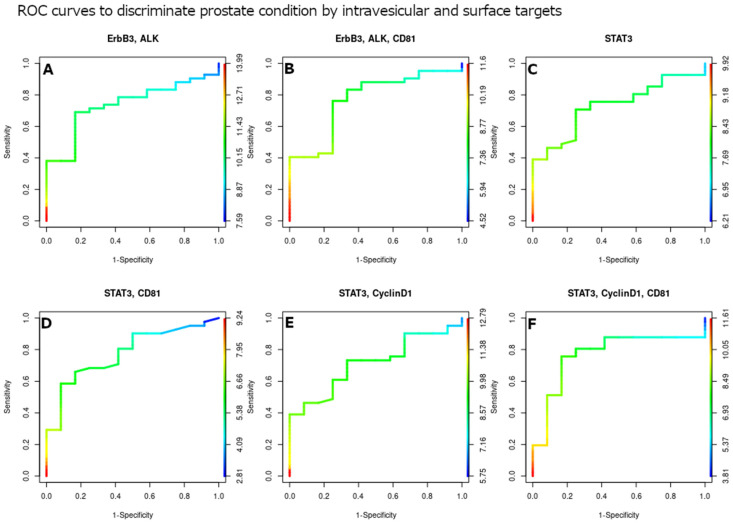
The receiver operating characteristic curves for the intra-vesicular and surface targets. ROC curves for intra-vesicular and surface targets with the most differentiated counts: ERBB3, ALK (**A**); ERBB3, ALK, and CD81 (**B**); STAT3 (**C**); STAT3 and CD81 (**D**); STAT3 and CyclinD1 (**E**); and STAT3, CyclinD1, and CD81 (**F**).

The power of the EV targets shown in Figure 3 to distinguish the tumor samples from the hyperplasia ones was studied using ROC curves (Figure 4). The number of EVs containing both STAT3 and CD81 had a higher area under the curve (AUC) of 0.78 (Figure 4D). The second wider region was that of STAT3 and CD81 with the presence of CyclinD1, where the AUC = 0.77 (Figure 4F). Instead STAT3 alone had an AUC = 0.73 (Figure 4C), and STAT3 with CyclinD1 had an AUC = 0.71 (Figure 4E). Similarly, some surface proteins are accurate in distinguishing prostate conditions; in particular, ERBB3 and ALK alone and in conjunction with CD81 show, respectively, AUCs of 0.74 and 0.77 (Figure 4A,B). We further evaluated the ability of PSA to classify PCa and BPH better than the other markers (Figure S6B), obtaining poor results (AUC = 0.67).

Table 3 shows the contingency tables for all the proteins of interest and their performance metrics associated with the selected threshold (Table S1 for non-discriminant ones). Consultation of Table 2 indicates that the best vesicular targets with the highest discriminating power between PCa and BPH is the combination of STAT3, CyclinD1, and CD81, which achieves the best performance in terms of the overall classification of positive and negative samples, as specified by the highest accuracy. Figure S3 shows the ROC curves for targets with similar counts in the two groups, which can be seen from the diagonal-like curve expressing no discrimination between the tumor and hyperplasia samples.

**Figure 5:**
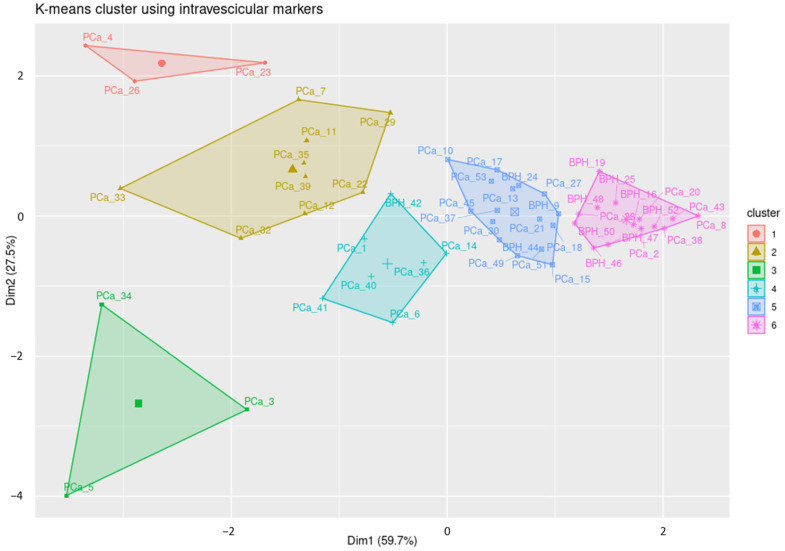
K-means cluster analysis. The above graph was generated from the analysis of the four most characterizing targets: STAT3; STAT3 and CD81; STAT3 and CyclinD1; and STAT3, CyclinD1, and CD81.

To better analyze the discriminative ability of the surface and intra-vesicular proteins, unsupervised cluster analysis was conducted using k-means to better visualize the similar groups. For the intra-vesicular dataset, Elbow’s method suggests six clusters, and Figure 5 shows their distribution. The first thing to note is the distance from the tumor samples to the hyperplasia samples. Cluster #6 had most of the hyperplasia samples, and a few of them also belong to cluster #5. The tumor samples however clustered into five different groups, which are likely to define a different clinical status, as discussed below. Furthermore, the variation in clinical characteristics seems to underline the aggregation of some BPH samples with the PCa clusters, and vice versa. Despite the clear influence, we cannot retrieve the clinical information to verify the correctness of our hypothesis.

### 3.3. sEV Protein Marker Correlation with Clinical Features

**Figure 6:**
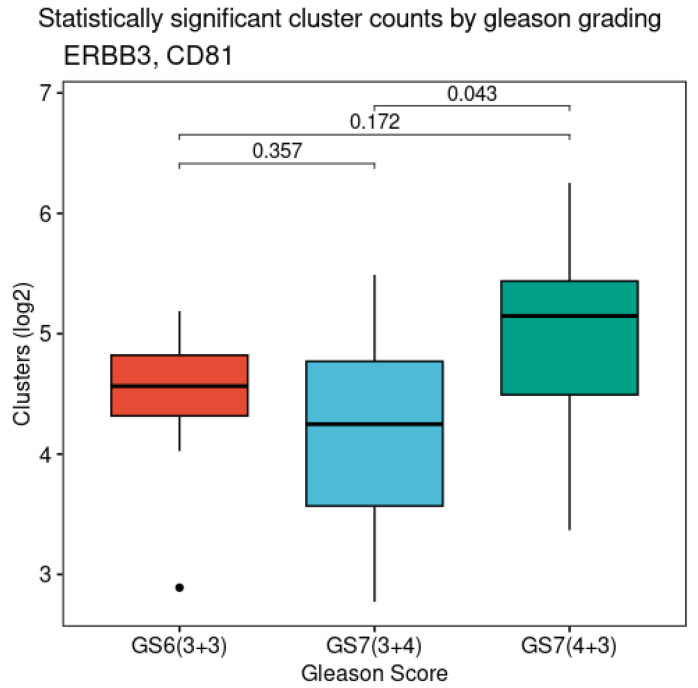
Cluster counts according to Gleason score for ERBB3 and CD81 markers. Boxplots show higher ERBB3 and CD81 levels in GS7(4 + 3) than those in GS7(3 + 4). These *p*-values were not adjusted due to small sample size. Symbol “•”represent values out of ±1.5 * IQR.

All the surface and intra-vesicular counts were also evaluated against clinical parameters such as the Gleason grade and cancer staging. A statistically significant difference was found in the ERBB3 and CD81 levels, showing a higher value in GS7(4 + 3) compared to that of the GS7(3 + 4) samples, *p* = 0.043 (Figure 6).

No other relevant differences were found in protein occurrences, varying the Gleason score (Figure S4). No significant differences were found even when PSA was considered as discriminant of the Gleason grade (Figure S6C).

Then, we assessed the relationship between cancer staging and the cluster counts without relevant findings. In the Supplementary Materials, Figure S5K,L shows the largest differences between the intra-vesicular and surface markers under study; the values of STAT3 and CyclinD1 in tumor stage 3a compared to those in stage 3b are statistically significant (*p* = 0.047), as is ERBB3 in combination with the ALK differences in expression levels between tumor stages 2c and 3a (*p* = 0.031). Finally, stage 2a has a higher level of expression than that of stage 3a, which is statistically significant (*p* = 0.049) for the ERBB3, ALK, and CD81 triplets. All the other markers show no statistically significant differences (Figure S5A–J), and this was the same for the PSA (Figure S6D).

**Figure 7:**
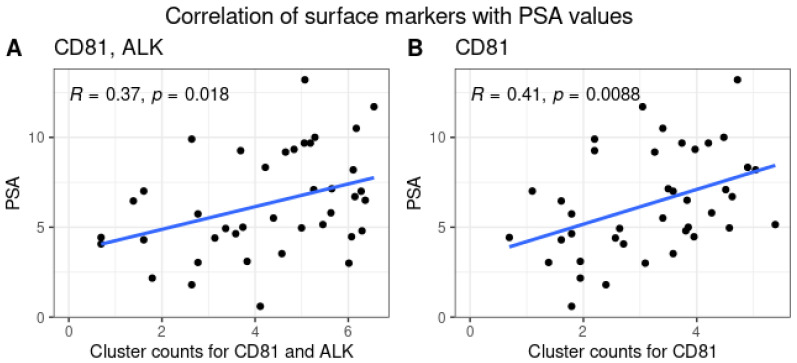
Correlation plots of surface markers with PSA values. The presented plots highlight the correlation between the PSA values and the cluster counts for both the CD81, ALK surface protein (**A**) and CD81 alone (**B**), showing a weak-to-moderate but statistically significant correlation.

Finally, the last parameter studied was the ratio of surface and intra-vesicular proteins with respect to the PSA. Analysis shows a statistically significant *p*-value with a weak-to-moderate correlation between the PSA values with those of CD81 alone and with ALK (Figure 7A,B).

## 4. Discussion

PSA testing is not suitable for population-based screening due to its low-level specificity for prostate cancer, and, in the last few years, it has resulted in misdiagnosis and consequent overtreatment.

When used for PCa screening in men aged between 50 and 74, 16% of individuals are positive for a high PSA level, but three out of four men with a high PSA level are cancer-negative according to biopsy [44]. This is a clear indication of a significant lack of specificity. Moreover, a low PSA result is not always associated with the absence of prostate cancer [45]. MRI has improved the current PCa diagnostic scenario, but the impact on PCa management is still limited.

Liquid biopsy (LB) is emerging as an exciting and revolutionary technology progressively navigating its way into clinical practice. Despite the existence of a plethora of potent and high-throughput tests, liquid biopsy’s capacity to significantly impact clinical management beyond mere prognostication for PCa remains somewhat constrained. The principal hurdle impeding LB’s seamless integration into clinical decision making lies in the low reliability of circulating molecular signals since copious amounts of genomic and transcriptomic data furnished by LB pose a formidable challenge in deciphering the meaningful signals amidst the background noise, thereby making the construction of this robust process a complex endeavor. However, as our understanding of integrating the knowledge concerning multiple concurrent molecular changes continues to evolve, the translation of this wealth of information into clinically actionable steps is anticipated to become increasingly attainable.

The points of greatest interest concern the possibility of a highly specific, minimally invasive test that has a predictive value equal to or greater than that of multiparametric magnetic resonance imaging (MRI). The ultimate, yet-to-be-achieved goal is to confidently diagnose the presence of prostate cancer without resorting to a prostate biopsy. Another unresolved point is how to predict the progression of a diagnosed tumor, especially in cases of lesions classified as Gleason 6, as such events are often considered clinically insignificant, and therefore are initiated into so-called active surveillance.

EVs have proven to be very interesting vehicles for molecular markers. Their important physiological function as carriers for long-distance signaling is utilized in pathological processes, playing key roles connected to tumor progression. This specific biological relevance makes them particularly significant, even when seeking specific signals of clinical interest for diagnostic or prognostic purposes. Furthermore, extracellular vesicles are particularly stable, and consequently, the molecular markers associated with them are theoretically less prone to modifications and fluctuations that could impact the reproducibility of measurements.

It is important to remember that although liquid biopsy through urine analysis is undoubtedly less invasive than blood-based biopsy, the latter is not affected by the concentration changes that the analyzed markers undergo during different times of the day. These variations, which are also subject to individual hydration conditions, can make the characterization of quantitative markers such as vesicular proteins complex.

Since EVs may reflect the molecular composition of the releasing cells, in this study, we assessed the feasibility of employing protein markers that are known to have an altered abundance in prostate cancer cells in blood-isolated EVs. Since the marker-related differences in patients with cancer and healthy individuals are quantitatively limited, and thus very specific, the importance of a very sensitive quantification method is fundamental. Super-resolution microscopy can evaluate the number of sEV expressing specific antibodies with really high precision and confidence, thus allowing for the powerful classification of samples according to their molecular profiles.

It has been shown that STAT3 is involved in the progression of prostate cancer [46].

Similarly, cyclinD1 has been repeatedly associated with the aggressiveness of prostate cancer and its tendency to cause bone metastases [47], while the overexpression of ALK has been observed in advanced prostate tumors [48]. The increase in these markers in sEVs of the patients with PCs compared to that of those individuals with BPH is consistent with those observations. On this basis, further studies will be necessary to evaluate whether these vesicular markers are predictive of tumor aggressiveness, and therefore, support the therapeutic strategy. In this study, for the first time, these specific tumor markers are evaluated in the sEVs of patients with prostate cancer using a sensitive and specific method, suggesting that there is a relationship between their biological role in tumor cells and their significant presence in vesicles.

While this study is limited to the classification of patients according to their diagnosis, the biology of the signature may be suggestive of potential applications for disease monitoring. This will require additional investigations on a different cohort of patients with longer follow-up specifically aimed at prognosis evaluation.

## 5. Conclusions

In this study, we explored, as a proof of concept, the possibility of utilizing the quantification of blood-derived sEV proteins, both internal and surface, to classify patients with PCa and BPH. The sEV markers were selected on the hypothesis that the cellular proteins that are known to be overexpressed in cancer may be overrepresented in the circulating sEVs population. This marks the first instance of employing super-resolution microscopy to develop such a liquid biopsy approach. The sensitivity and robustness of measurement, along with the obtained results, suggest that this technology could be adapted for routine and sensitive applications, such as the differential diagnosis of prostate cancer. Although the limited case series prevents the exact estimation of the accuracy of the proposed application system, the data indicate that the quantitative combination of multiple protein markers enhances the classification power of the method. Moreover, the ability to utilize very small sample volumes suggests that the technique may offer specific advantages in the protocols, requiring repeated sampling.

## Figures and Tables

**Table 1:** List of antibodies used in WB.

Antibody	Dilution	Brand	Catalog Number
Mouse monoclonal anti-CD45	1:500	R&D Systems	MAB14303
Mouse monoclonal anti-Alix (3A9)	1:1000	Cell Signaling Technology	2171S
Mouse monoclonal anti-β-Actin	1:10,000	Sigma Aldrich	A1978
HRP-conjugated anti-mouse secondary antibody	1:10,000	Dako	P0447

**Table 2:** Patients’ clinic characteristics.

Total Samples		N
	Prostate Adenocarcinoma (PCa)	42
	Benign Prostate Hyperplasia (BPH)	12
GLEASON SCORE		
	6 (3 + 3)	11
	7 (3 + 4)	16
	7 (4 + 3)	11
	Not recorded	4
STAGING		
	T2a	6
	T2b	1
	T2c	20
	T3a	4
	T3b	5
	N0	10
	Nx	18
	N1	4
	T-N not recorded	6
	M0	1
	M1	0
	Mx	13
	M not recorded	28
PSA		MEDIAN (IQR)
	total	6.46 (4.43–9.26)
	PCa	6.60 (4.51–9.24)
	BPH	5 (3.07–8.18)
AGE		MEDIAN (IQR)
	total	67.50 (63–72.75)
	PCa	67.50 (62.25–72.75)
	BPH	67.50 (65.25–72.25)

**Table 3:** Contingency tables with cutoff values and performance metrics for markers: ERBB3 and ALK; ERBB3, ALK, and CD81; STAT3; STAT3 and CD81; STAT3 and CyclinD1; and STAT3, CyclinD1, and CD81.

	PCa	BPH	Sensitivity	Specificity	Accuracy
ERBB3, ALK ≥ 10.03	30	3	0.75	0.71	0.72
ERBB3, ALK < 10.03	12	9			
ERBB3, ALK, CD81 ≥ 8.15	31	3	0.75	0.73	0.74
ERBB3, ALK, CD81 < 8.15	11	9			
STAT3 ≥ 8.07	29	3	0.75	0.70	0.71
STAT3 < 8.07	12	9			
STAT3, CD81 ≥ 5.72	28	3	0.75	0.68	0.69
STAT3, CD81 < 5.72	13	9			
STAT3, CyclinD1 ≥ 9.36	27	4	0.66	0.65	0.66
STAT3, CyclinD1 < 9.36	14	8			
STAT3, CyclinD1, CD81 ≥ 8.08	31	2	0.83	0.75	0.77
STAT3, CyclinD1, CD81 < 8.08	10	10			

## Data Availability

EV counting raw data generated by CODI are provided as Supplementary Materials (EV_counting_dataset.zip).

## Supplementary Materials

The following supporting information can be downloaded at: https://www.mdpi.com/article/10.3390/cells13151286/s1, Figure S1: Super-resolution microscopy-based EV characterization with markers not showing significant differences; Figure S2: Intra-vesicular and surface markers without statistically significant differences between neoplastic conditions; Figure S3: Receiver operating characteristics curves for intra-vesicular and surface markers not statistically significant; Table S1: Contingency table with cutoff values and performance metrics for markers: ALK; CD81; CD81 and ALK; CD81 and CyclinD1; CyclinD1; ERBB3; ERBB3, and CD81; Figure S4: Cluster counts for Gleason classification (not statistically significant markers); Figure S5: Cluster counts grouped by tumor staging for intra-vesicular and surface markers; Figure S6: PSA values related to all clinical conditions under study; Dataset S1: EV counting raw data generated by CODI.

## References

[1] Global Cancer Observatory (GCO) of the International Agency for Research on Cancer (IARC) Cancer Today. Prostate Cancer Factsheet. https://gco.iarc.fr/today/data/factsheets/cancers/27-Prostate-fact-sheet.pdf.

[2] Siegel R.L., Miller K.D., Jemal A. (2020). Cancer statistics. CA Cancer J. Clin..

[3] Gandaglia G., Bray F., Cooperberg M.R., Leveridge M.J., Moretti K., Murphy D.G., Penson D.F., Miller D.C. (2016). Prostate Cancer Registries: Current Status and Future Directions. Eur. Urol..

[4] Moyer V.A., U.S. Preventive Services Task Force (2012). Screening for prostate cancer: U.S. Preventive Services Task Force recommendation statement. Ann. Intern. Med..

[5] Pinsky P.F., Prorok P.C., Yu K., Kramer B.S., Black A., Gohagan J.K., Crawford E.D., Grubb R.L., Andriole G.L. (2017). Extended mortality results for prostate cancer screening in the PLCO trial with median follow-up of 15 years. Cancer.

[6] Catalona W.J., Smith D.S., Ratliff T.L., Dodds K.M., Coplen D.E., Yuan J.J., Petros J.A., Andriole G.L. (1991). Measurement of prostate-specific antigen in serum as a screening test for prostate cancer. N. Engl. J. Med..

[7] Schröder F.H., Hugosson J., Roobol M.J., Tammela T.L., Ciatto S., Nelen V., Kwiatkowski M., Lujan M., Lilja H., Zappa M. (2009). Screening and prostate-cancer mortality in a randomized European study. N. Engl. J. Med..

[8] Loeb S., Bjurlin M.A., Nicholson J., Tammela T.L., Penson D.F., Carter H.B., Carroll P., Etzioni R. (2014). Overdiagnosis and overtreatment of prostate cancer. Eur. Urol..

[9] Institute for Quality and Efficiency in Health Care (IQWiG) (2019). Prostate Cancer Screening with the PSA Test: Preliminary Report.

[10] Siddiqui M.M., Rais-Bahrami S., Turkbey B., George A.K., Rothwax J., Shakir N., Okoro C., Raskolnikov D., Parnes H.L., Linehan W.M. (2015). Comparison of MR/ultrasound fusion-guided biopsy with ultrasound-guided biopsy for the diagnosis of prostate cancer. JAMA.

[11] Vargas H.A., Hötker A.M., Goldman D.A., Moskowitz C.S., Gondo T., Matsumoto K., Ehdaie B., Woo S., Fine S.W., Reuter V.E. (2016). Updated prostate imaging reporting and data system (PI-RADS v2) recommendations for the detection of clinically significant prostate cancer using multiparametric MRI: Critical evaluation using whole-mount pathology as standard of reference. Eur. Radiol..

[12] Sonn G.A., Fan R.E., Ghanouni P., Wang N.N., Brooks J.D., Loening A.M., Daniel B.L., To’o K.J., Thong A.E., Leppert J.T. (2019). Prostate Magnetic Resonance Imaging Interpretation Varies Substantially Across Radiologists. Eur. Urol. Focus.

[13] Cooperberg M.R., Carroll P.R. (2015). Trends in Management for Patients with Localized Prostate Cancer, 1990–2013. JAMA.

[14] Rappa G., Puglisi C., Santos M.F., Forte S., Memeo L., Lorico A. (2019). Extracellular Vesicles from Thyroid Carcinoma: The New Frontier of Liquid Biopsy. Int. J. Mol. Sci..

[15] McKiernan J., Donovan M.J., O’Neill V., Bentink S., Noerholm M., Belzer S., Skog J., Kattan M.W., Partin A., Andriole G. (2016). A Novel Urine Exosome Gene Expression Assay to Predict High-grade Prostate Cancer at Initial Biopsy. JAMA Oncol..

[16] Nilsson J., Skog J., Nordstrand A., Baranov V., Mincheva-Nilsson L., Breakefield X.O., Widmark A. (2009). Prostate cancer-derived urine exosomes: A novel approach to biomarkers for prostate cancer. Br. J. Cancer.

[17] Bryant R.J., Pawlowski T., Catto J.W., Marsden G., Vessella R.L., Rhees B., Kuslich C., Viskorpi T., Hamdy F.C. (2012). Changes in circulating microRNA levels associated with prostate cancer. Br. J. Cancer.

[18] Duijvesz D., Burnum-Johnson K.E., Gritsenko M.A., Hoogland A.M., Vredenbregt-van den Berg M.S., Willemsen R., Luider T., Paša-Tolić L., Jenster G. (2013). Proteomic Profiling of Exosomes Leads to the Identification of Novel Biomarkers for Prostate Cancer. PLoS ONE.

[19] Trujillo B., Wu A., Wetterskog D., Attard G. (2022). Blood-based liquid biopsies for prostate cancer: Clinical opportunities and challenges. Br. J. Cancer.

[20] Oshi M., Murthy V., Takahashi H., Huyser M., Okano M., Tokumaru Y., Rashid O.M., Matsyama R., Endo I., Takabe K. (2021). Urine as a Source of Liquid Biopsy for Cancer. Cancers.

[21] Halvaei S., Daryani S., Eslami-S Z., Samadi T., Jafarbeik-Iravani N., Bakhshayesh T.O., Majidzadeh-A K., Esmaeili R. (2018). Exosomes in Cancer Liquid Biopsy: A Focus on Breast Cancer. Mol. Ther. Nucleic Acids.

[22] Raposo G., Stoorvogel W. (2013). Extracellular vesicles: Exosomes, microvesicles, and friends. J. Cell Biol..

[23] Théry C., Witwer K.W., Aikawa E., Alcaraz M.J., Anderson J.D., Andriantsitohaina R., Antoniou A., Arab T., Archer F., Atkin-Smith G.K. (2018). Minimal information for studies of extracellular vesicles 2018 (MISEV2018): A position statement of the International Society for Extracellular Vesicles and update of the MISEV2014 guidelines. J. Extracell. Vesicles.

[24] Craft N., Shostak Y., Carey M., Sawyers C.L. (1999). A mechanism for hormone-independent prostate cancer through modulation of androgen receptor signaling by the HER-2/neu tyrosine kinase. Nat. Med..

[25] Molife L.R., Omlin A., Jones R.J., Karavasilis V., Bloomfield D., Lumsden G., Fong P.C., Olmos D., O’Sullivan J.M., Pedley I. (2014). Randomized Phase II trial of nintedanib, afatinib and sequential combination in castration-resistant prostate cancer. Future Oncol..

[26] Koumakpayi I.H., Diallo J.S., Le Page C., Lessard L., Gleave M., Bégin L.R., Mes-Masson A.M., Saad F. (2006). Expression and nuclear localization of ErbB3 in prostate cancer. Clin. Cancer Res..

[27] Carneiro B.A., Pamarthy S., Shah A.N., Sagar V., Unno K., Han H., Yang X.J., Costa R.B., Nagy R.J., Lanman R.B. (2018). Anaplastic lymphoma kinase mutation (ALK F1174C) in small cell carcinoma of the prostate and molecular response to alectinib. Clin. Cancer Res..

[28] Dulińska-Litewka J., Felkle D., Dykas K., Handziuk Z., Krzysztofik M., Gąsiorkiewicz B. (2022). The role of cyclins in the development and progression of prostate cancer. Biomed. Pharmacother..

[29] Gu L., Dagvadorj A., Lutz J., Leiby B., Bonuccelli G., Lisanti M.P., Addya S., Fortina P., Dasgupta A., Hyslop T. (2010). Transcription factor Stat3 stimulates metastatic behavior of human prostate cancer cells in vivo, whereas Stat5b has a preferential role in the promotion of prostate cancer cell viability and tumor growth. Am. J. Pathol..

[30] Mazzocca A., Liotta F., Carloni V. (2008). Tetraspanin CD81-regulated cell motility plays a critical role in intrahepatic metastasis of hepatocellular carcinoma. Gastroenterology.

[31] Yoo T.H., Ryu B.K., Lee M.G., Chi S.G. (2013). *CD81* is a candidate tumor suppressor gene in human gastric cancer. Cell. Oncol..

[32] Luga V., Zhang L., Viloria-Petit A.M., Ogunjimi A.A., Inanlou M.R., Chiu E., Buchanan M., Hosein A.N., Basik M., Wrana J.L. (2012). Exosomes mediate stromal mobilization of autocrine Wnt-PCP signaling in breast cancer cell migration. Cell.

[33] Vences-Catalán F., Rajapaksa R., Kuo C.C., Miller C.L., Lee A., Ramani V.C., Jeffrey S.S., Levy R., Levy S. (2021). Targeting the tetraspanin CD81 reduces cancer invasion and metastasis. Proc. Natl. Acad. Sci. USA.

[34] Uretmen Kagiali Z.C., Sanal E., Karayel Ö., Polat A.N., Saatci Ö., Ersan P.G., Trappe K., Renard B.Y., Önder T.T., Tuncbag N. (2019). Systems-level Analysis Reveals Multiple Modulators of Epithelial-mesenchymal Transition and Identifies DNAJB4 and CD81 as Novel Metastasis Inducers in Breast Cancer. Mol. Cell Proteom..

[35] Mizoshiri N., Shirai T., Terauchi R., Tsuchida S., Mori Y., Hayashi D., Kishida T., Arai Y., Mazda O., Nakanishi T. (2019). The tetraspanin CD81 mediates the growth and metastases of human osteosarcoma. Cell. Oncol..

[36] Hong I.K., Byun H.J., Lee J., Jin Y.J., Wang S.J., Jeoung D.I., Kim Y.M., Lee H. (2014). The tetraspanin CD81 protein increases melanoma cell motility by up-regulating metalloproteinase MT1-MMP expression through the pro-oncogenic Akt-dependent Sp1 activation signaling pathways. J. Biol. Chem..

[37] Zhang Y., Qian H., Xu A., Yang G. (2020). Increased expression of *CD81* is associated with poor prognosis of prostate cancer and increases the progression of prostate cancer cells in vitro. Exp. Ther. Med..

[38] Leggio L., L’Episcopo F., Magrì A., Ulloa-Navas M.J., Paternò G., Vivarelli S., Bastos C.A.P., Tirolo C., Testa N., Caniglia S. (2022). Small Extracellular Vesicles Secreted by Nigrostriatal Astrocytes Rescue Cell Death and Preserve Mitochondrial Function in Parkinson’s Disease. Adv. Healthc. Mater..

[39] R Core Team (2014). A Language and Environment for Statistical Computing.

[40] R Studio Team (2021). Integrated Development Environment for R.

[41] Sing T., Sander O., Beerenwinkel N., Lengauer T. (2005). ROCR: Visualizing classifier performance in R. Bioinformatics.

[42] Kassambara A. (2023). ggpubr: ‘ggplot2’ Based Publication Ready Plots. https://rpkgs.datanovia.com/ggpubr/.

[43] Wickham H. (2016). ggplot2: Elegant Graphics for Data Analysis.

[44] Schröder F.H., Hugosson J., Roobol M.J., Tammela T.L., Ciatto S., Nelen V., Kwiatkowski M., Lujan M., Lilja H., Zappa M. (2012). ERSPC Investigators. Prostate-cancer mortality at 11 years of follow-up. N. Engl. J. Med..

[45] Croswell J.M., Kramer B.S., Crawford E.D. (2011). Screening for prostate cancer with PSA testing: Current status and future directions. Oncology.

[46] Abdulghani J., Gu L., Dagvadorj A., Lutz J., Leiby B., Bonuccelli G., Lisanti M.P., Zellweger T., Alanen K., Mirtti T. (2008). Stat3 promotes metastatic progression of prostate cancer. Am. J. Pathol..

[47] Drobnjak M., Osman I., Scher H.I., Fazzari M., Cordon-Cardo C. (2000). Overexpression of cyclin D1 is associated with metastatic prostate cancer to bone. Clin. Cancer Res..

[48] Patel R.A., Coleman I., Roudier M.P., Konnick E.Q., Hanratty B., Dumpit R., Lucas J.M., Ang L.S., Low J.Y., Tretiakova M.S. (2022). Comprehensive assessment of anaplastic lymphoma kinase in localized and metastatic prostate cancer reveals targetable alterations. Cancer Res. Commun..

