# Supplementary material for: A Novel Liquid Biopsy Method Based on Specific Combinations of Vesicular Markers Allows Us to Discriminate Prostate Cancer from Hyperplasia
Source: Cells. 2024 Jul 31;13(15):1286. doi: 10.3390/cells13151286 (PMC11311686; doi:10.3390/cells13151286)
Supplement: Supplementary file 1 [file cells-13-01286-s001.zip › cells-3002803-supplementary.pdf]

Supplementary material

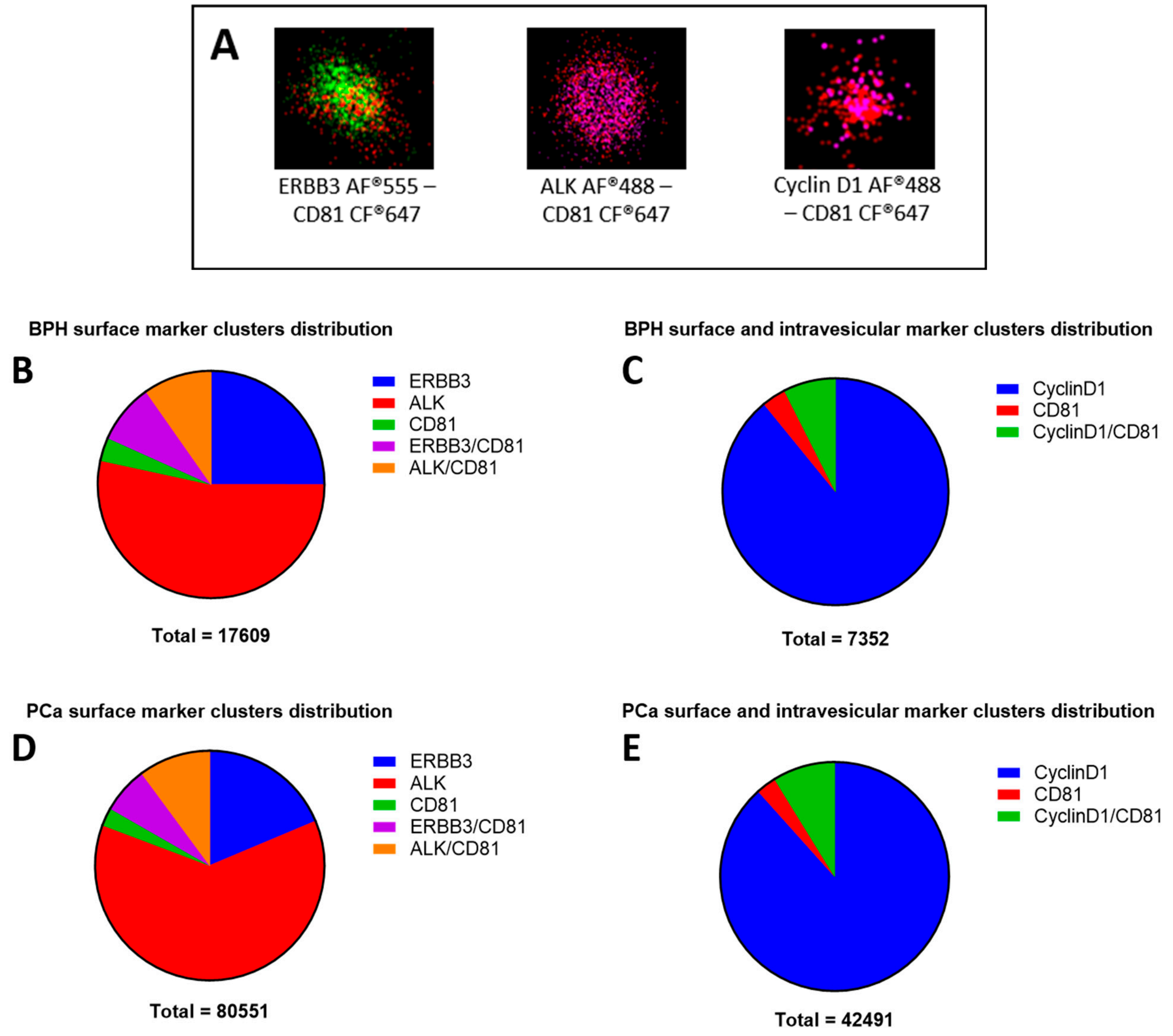

F

	PCa	BPH
**ERBB3**	15049	4410
**ALK**	49981	9394
**CD81**	2046	585
**ERBB3/CD81**	5311	1502
**ALK/CD81**	8164	1718
**Total**	80551	17609

G

	PCa	BPH
**CyclinD1**	37543	6549
**CD81**	1209	253
**CyclinD1/CD81**	3739	550
**Total**	42491	7352

Figure S1: Super-resolution microscopy-based EV characterization with markers not showing significant differences. Representative images of single sEVs contemporarily co-expressing two markers of those ones not considered in the following experiments (A). Pie charts of BPH surface-only (B) and surface/intravesicular (C) markers distribution and of PCa surface-only (D) and surface/intravesicular (E) ones, which resulted not significant in the subsequent experiments. Not relevant surface-only (F) and surface/intravesicular (G) cluster counts for both types of samples. Abbreviations: sEVs: small extracellular vesicles; PCa: prostate cancer; BPH: benign prostate hyperplasia.

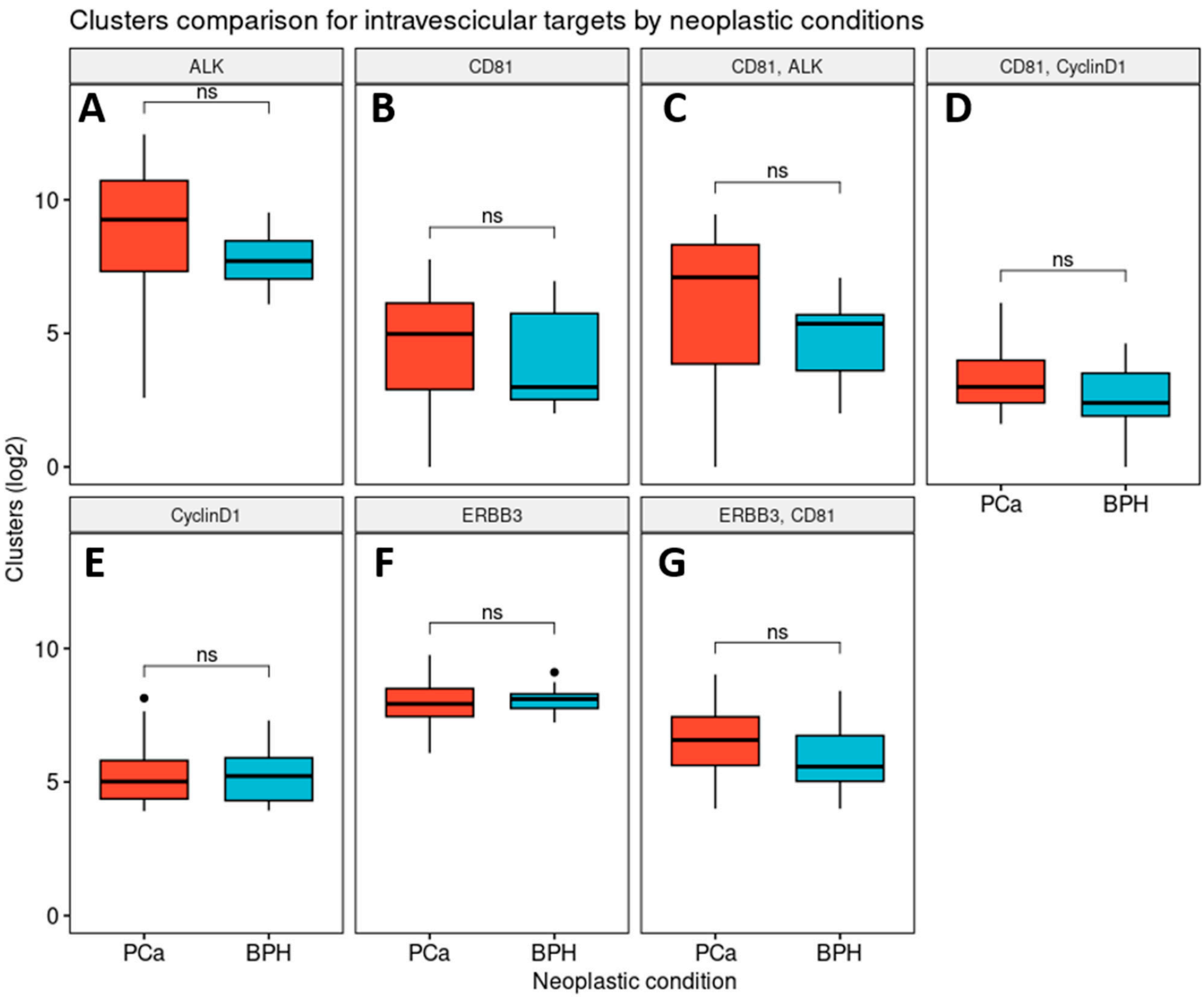

Figure S2: Intravesicular and surface markers without statistically significant differences between neoplastic conditions.

ROC curves to discriminate prostate condition by intravesicular and surface targets

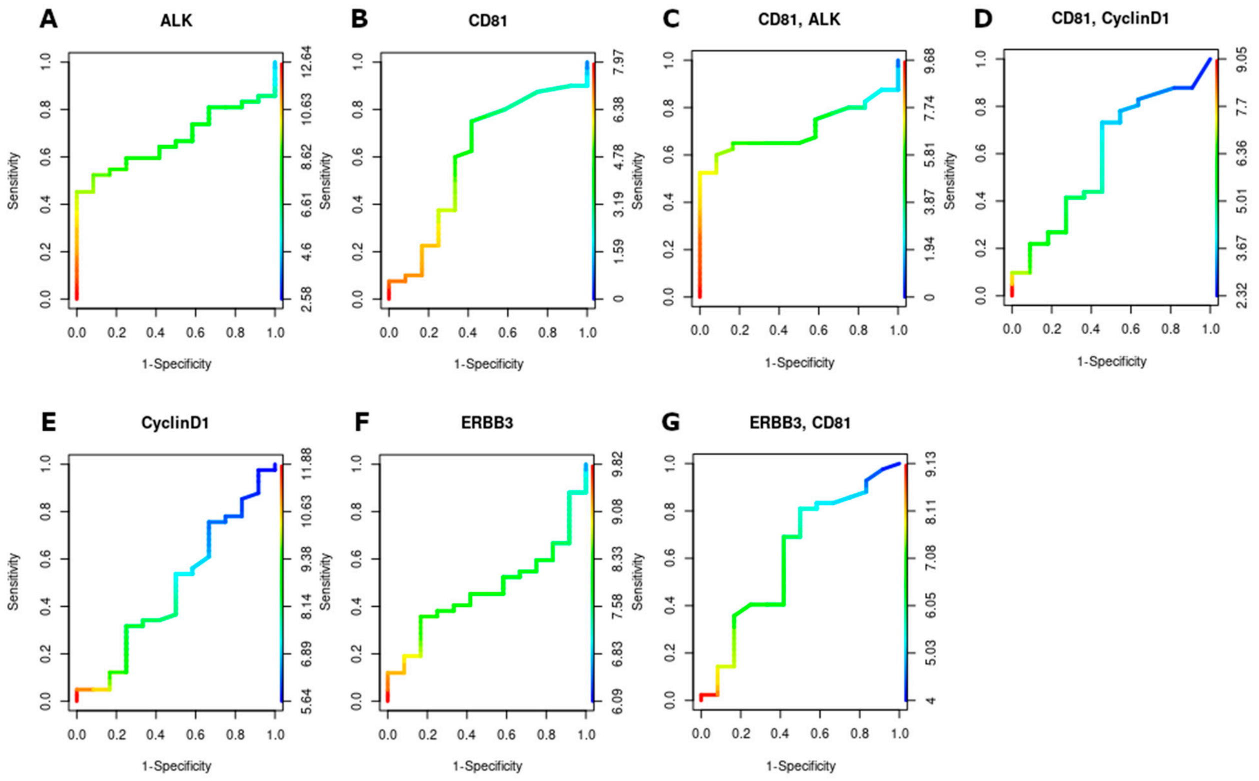

Figure S3: Receiver operating characteristics curves for intravesicular and surface markers not statistically significant. ROC curves show their inaccuracy to discriminate: ALK AUC=0.67 (Fig. S3A); CD81 has an AUC=0.60 (Fig. S3B); CD81 and ALK have AUC=0.70 (Fig. S3C); CD81 and CyclinD1 AUC=0.59 (Fig. S3D); CyclinD1 AUC=0.47 (Fig. S3E). ERBB3 AUC=0.46 (Fig. S3F); ERBB3, CD81 AUC=0.61 (Fig. S3G).

	PCa	BPH	Sensitivity	Specificity	Accuracy
**ALK ≥ 8.48**	25	3	0.75	0.59	0.62
**ALK < 8.48**	17	9			
**CD81 ≥ 4.70**	24	4	0.66	0.57	0.59
**CD81 < 4.70**	18	8			
**CD81, ALK ≥ 5.61**	26	4	0.66	0.61	0.62
**CD81, ALK < 5.61**	16	8			
**CD81, CyclinD1 ≥ 4.39**	20	5	0.58	0.48	0.50
**CD81, CyclinD1 < 4.39**	21	7			
**CyclinD1 ≥ 7.41**	20	6	0.50	0.48	0.49
**CyclinD1 < 7.41**	21	6			
**ERBB3 ≥ 8.16**	19	6	0.50	0.45	0.46
**ERBB3 < 8.16**	23	6			
**ERBB3, CD81 ≥ 6.40**	24	5	0.58	0.57	0.57
**ERBB3, CD81 < 6.40**	18	7			

Table S1: Contingency table with cutoff values and performance metrics for markers: ALK; CD81; CD81, ALK; CD81, CyclinD1; CyclinD1; ERBB3; ERBB3, CD81.

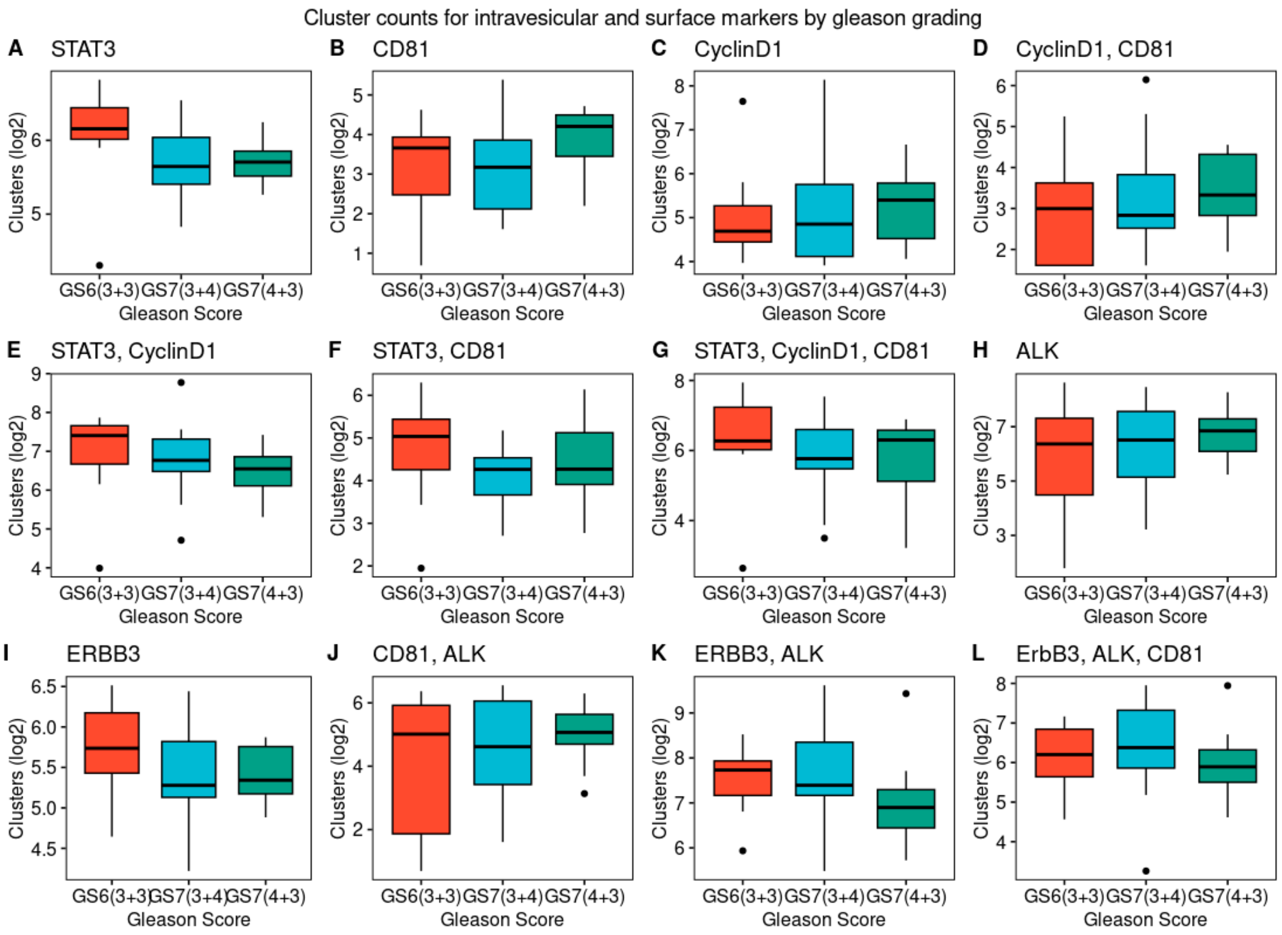

Figure S4: Cluster counts for Gleason classification (not statistically significant markers).

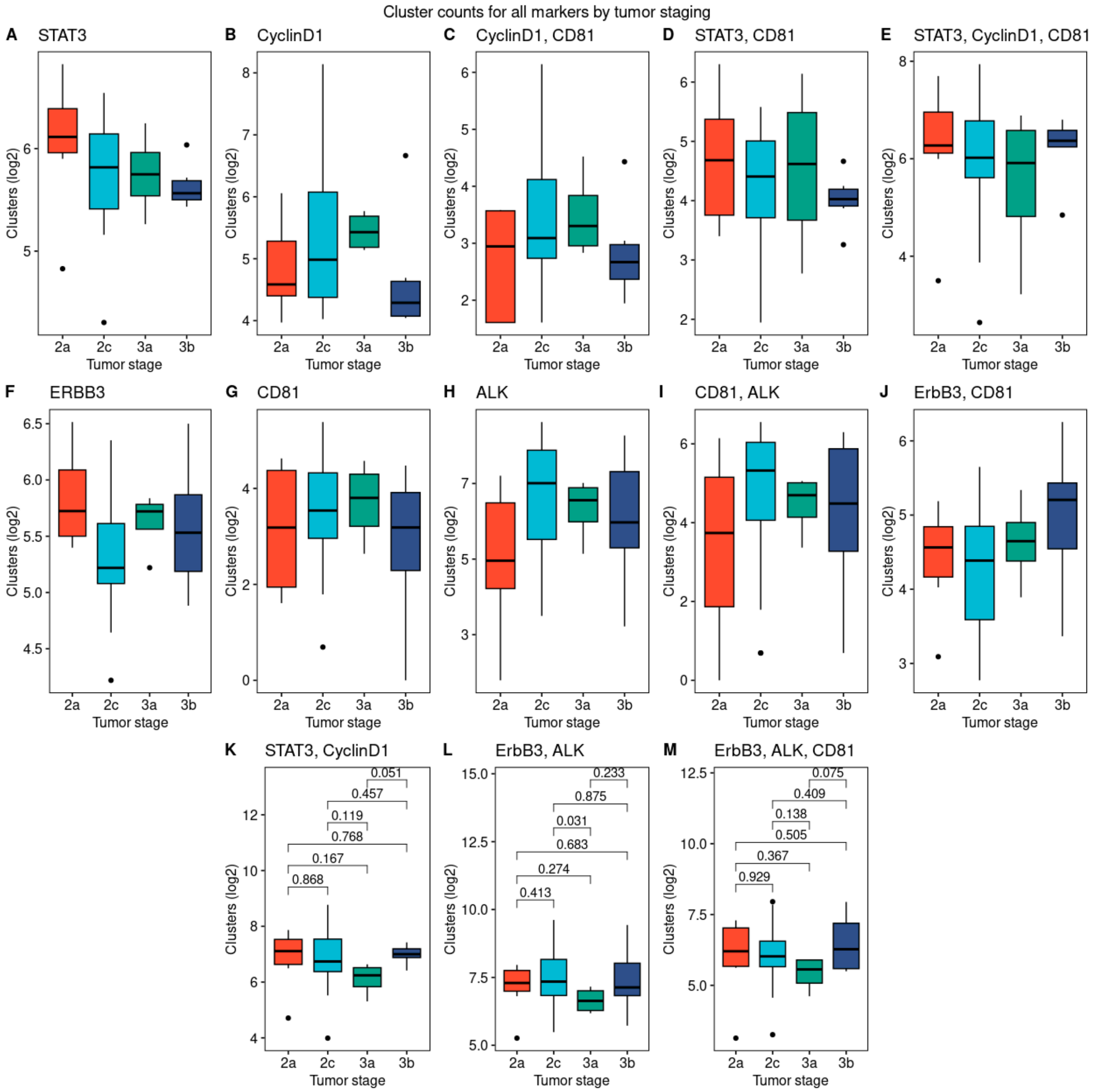

Figure S5: Cluster counts grouped by tumor staging for intravesicular and surface markers. STAT3, CyclinD1 (Fig. S5K) shows statistically significant difference in stage 3a versus 3b; in Fig. S5L ErbB3 and ALK have a higher value in stage 2c than in 3a; ErbB3, ALK and CD81 (Fig. S5M) show a relevant discrepancy in stage 2a compared to 3a counts. STAT3 (Fig. S5A) in tumor stage 2a vs 3b should be mentioned for discrepant but not statistically significant counts due to outlier.

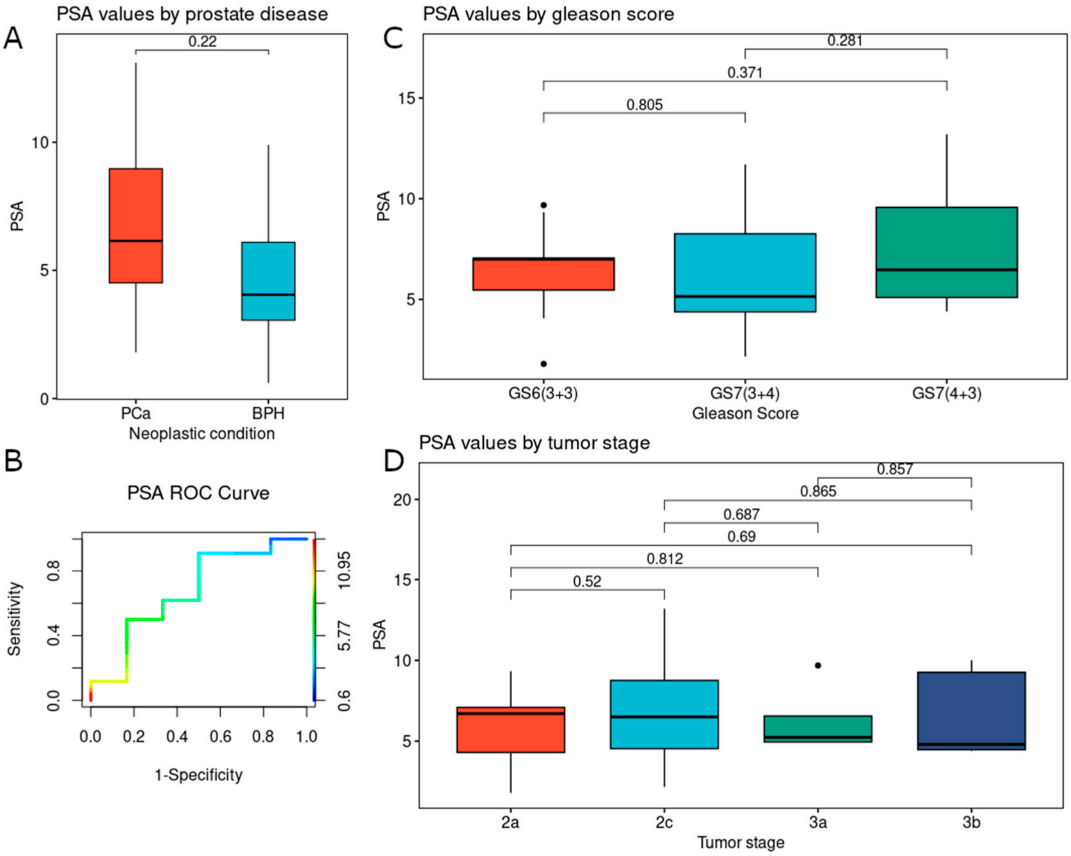

Figure S6: Evaluation of PSA values in different clinical conditions: Fig. S6A, boxplot showing the distribution of PSA values between PCa and BPH; Fig. S6B, ROC curve for PSA to discriminate between PCa and BPH; Fig. S6C, differences in PSA values grouping by gleason grading; Fig. S6D, distribution of PSA values by tumor stage under study.
